# 4D flow measurements in the superior cerebellar artery at 7 Tesla: feasibility and potential for applications in patients with trigeminal neuralgia

**DOI:** 10.1186/1532-429X-15-S1-W21

**Published:** 2013-01-30

**Authors:** S Schmitter, BD Jagadeesan, AW Grande, J Sein, K Ugurbil, P Van de Moortele

**Affiliations:** 1 Center for Magnetic Resonance Research, University of Minnesota, Minneapolis, MN, USA; 2 Departments of Radiology and Neurosurgery, University of Minnesota, Minneapolis, MN, USA; 3 Departments of Radiology, Neurosurgery and Neurology, University of Minnesota, Minneapolis, MN, USA

## Background

Trigeminal Neuralgia is characterized by episodic severe facial pain possibly the result of “pulsatile” compression of the nerve Root Entry Zone (REZ) of the Trigeminal Nerve (TN) by the adjacent superior cerebellar artery (SCA). The exact role of pulsatility of SCA remains unknown and currently no methods are available to measure the pulsatility of the SCA. Herein we investigate the feasibility of measuring blood flow and the Pulsatility Index (PI) within SCA and within the nearby posterior cerebral artery (PCA) using 4D-flow in a normal volunteer at 7T.

## Methods

**Figure 1:**
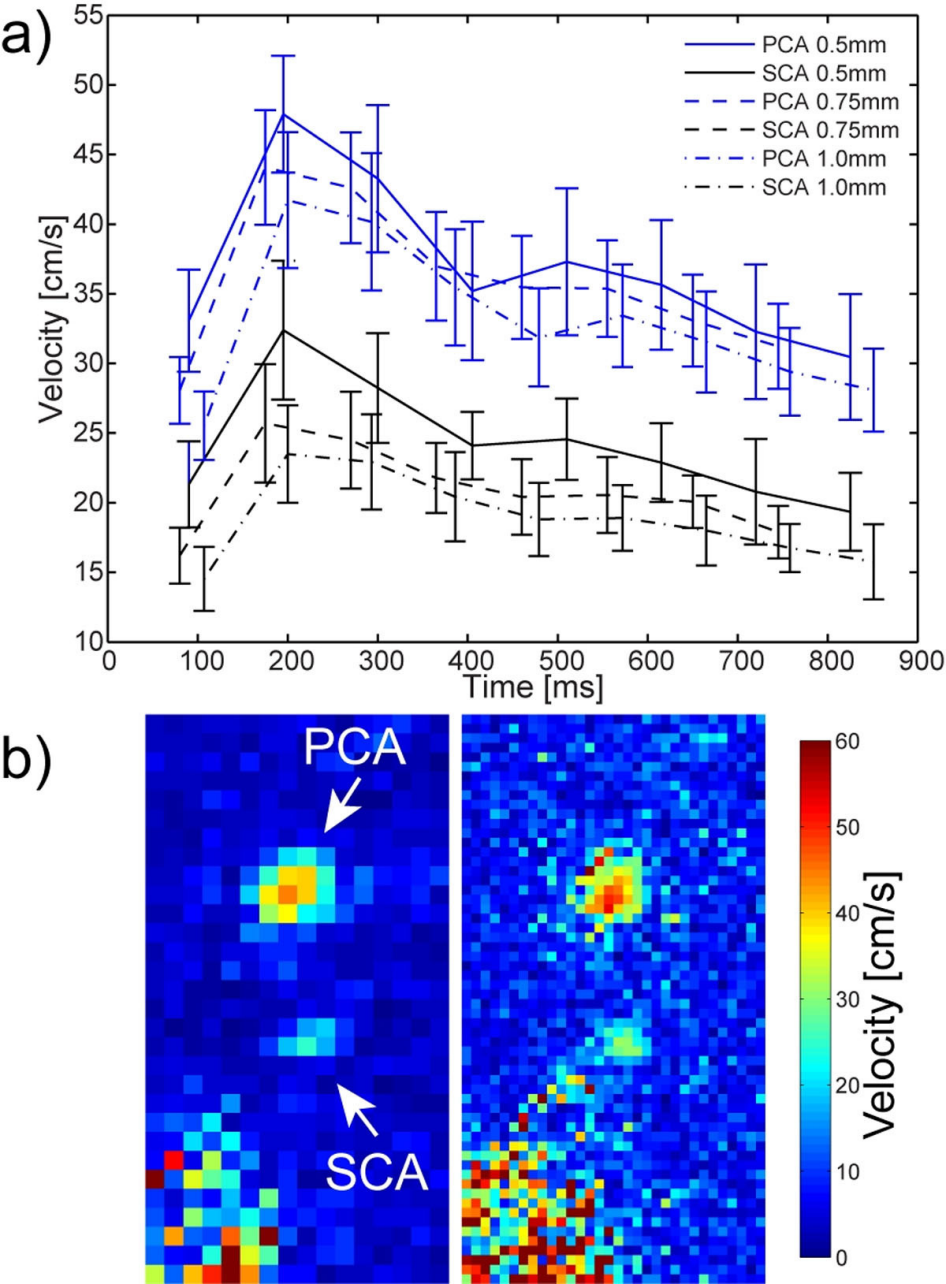
a) Velocities within the center of PCA and SCA averaged over 15 slices as a function of the cardiac phase. b) Velocity maps acquired in perpendicular orientation to SCA and PCA with 1mm (left) and 0.5mm (right) inplane resolution

Data were collected at 7T (Siemens,Germany) in a healthy 27 year-old male. T1w 3D-MPRAGE data was acquired to identify the the TN (see Fig.1a). For localization of SCA/PCA and planning of the 4D-flow acquisition, a (0.4mm)3 resolution TOF dataset was acquired (Fig.1b+c) with TR/TE=25/2.4ms,FA=22. Three 4D-flow acquisitions were performed perpendicular to the SCA (indicated in Fig.1c) with constant (1mm) slice thickness but with isotropic in-plane resolutions of 1mm/0.75mm/0.5mm. Parameters: VENC=60cm/s;FA=12°;TR=105/95/93ms (for 0.5/0.75/1mm resolution respectively); TE=4.5/4.0/4.0ms;8/8/9 cardiac phases. Post-processing, including correction of linear phase shifts determined within static tissue, was performed in Matlab. No spatial filtering was applied. The center voxel positions of PCA&SCA (one per slice and artery) were identified along 15 contiguous slices in magnitude images. Averaged over those 2x15 posititions, the time resolved velocity (and standard deviation) was computed within the phase-difference datasets. PIs were computed from the temporal curves using (vmax in systole) - vmin in diastole))/vmean.

## Results

**Figure 2:**
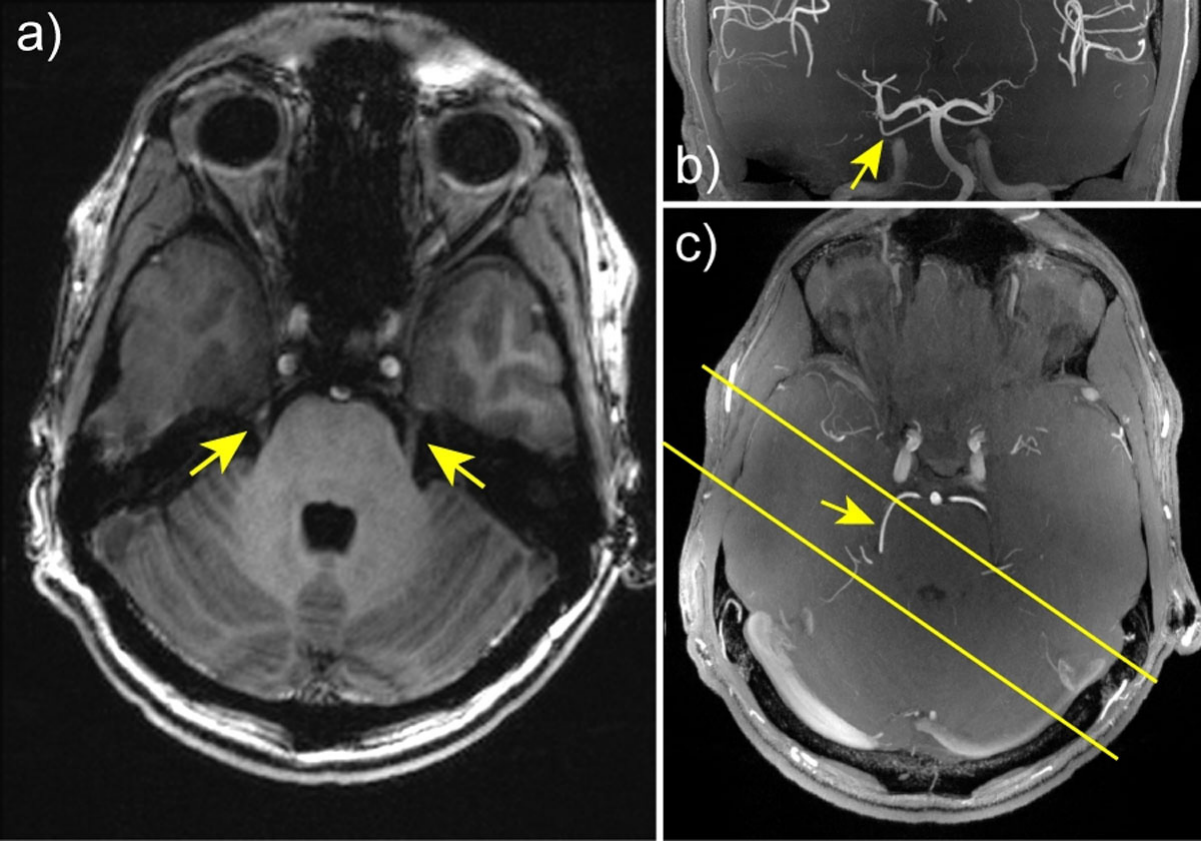
a) T1w MPRAGE image showing the Trigeminal Neve; b+c) axial and coronal MIP views of high resolution TOF images used for positioning and identifying the SCA (arrow). Yellow bars indicate the acquired slab for 4D flow acquisition.

Fig.1a clearly outlines the TN, located inferior to SCA. Axial+coronal MIPs shown in Fig.1b+c highlight the strong TOF contrast at 7T (signal ratios of ~4 between SCA and background tissue). Fig.2a shows mean velocity (+/-std) curves within the center of SCA (black) and PCA (blue) for different resolutions. Significant velocity difference between PCA and SCA is visible for all resolutions with systolic peak values of 47.9cm/s and 32.4cm/s (for 0.5mm). The PIs amount to 0.54/0.45/0.47 (SCA) and 0.47/0.45/0.49 (PCA) for 0.5/0.75/1mm resolutions. Importantly, a consistent trend towards lower velocities with increasing voxel size is visible for both arteries, likely due to partial volume effects. Consistent with this observation, 2D velocity maps perpendicular to the arteries (Fig.2b) clearly reflect the loss of details within SCA at 1mm vs. 0.5mm resolution.

## Conclusions

We demonstrated the feasibility of measuring velocity and PIs within SCA using 4D-flow at 7T. We also show that the measured values are directly affected by in-plane resolution. Comparisons of the PIs and velocity values within SCA loops in patients and normal people may help understanding the role of “pulsatile” compression in Trigeminal Neuralgia.

## Funding

P41 EB015894, S10 RR026783, R21-EB009138, KECK Foundation

